# Progressive Protrusive Tongue Exercise Does Not Alter Aging Effects in Retrusive Tongue Muscles

**DOI:** 10.3389/fphys.2021.740876

**Published:** 2021-10-21

**Authors:** Tiffany J. Glass, Joanie E. Figueroa, John A. Russell, Brittany N. Krekeler, Nadine P. Connor

**Affiliations:** ^1^Department of Surgery-Otolaryngology, University of Wisconsin-Madison, Madison, WI, United States; ^2^San Juan Bautista School of Medicine, Caguas, PR, United States; ^3^Department of Communication Sciences and Disorders, Northwestern University, Evanston, IL, United States; ^4^Department of Communication Sciences and Disorders, University of Wisconsin-Madison, Madison, WI, United States

**Keywords:** tongue exercise, hyoglossus, styloglossus, rat, aging, muscle

## Abstract

**Purpose:** Exercise-based treatment approaches for dysphagia may improve swallow function in part by inducing adaptive changes to muscles involved in swallowing and deglutition. We have previously shown that both aging and progressive resistance tongue exercise, in a rat model, can induce biological changes in the genioglossus (GG); a muscle that elevates and protrudes the tongue. However, the impacts of progressive resistance tongue exercise on the retrusive muscles (styloglossus, SG; hyoglossus, HG) of the tongue are unknown. The purpose of this study was to examine the impact of a progressive resistance tongue exercise regimen on the retrusive tongue musculature in the context of aging. Given that aging alters retrusive tongue muscles to more slowly contracting fiber types, we hypothesized that these biological changes may be mitigated by tongue exercise.

**Methods:** Hyoglossus (HG) and styloglossus (SG) muscles of male Fischer 344/Brown Norway rats were assayed in age groups of young (9 months old, *n* = 24), middle-aged (24 months old, *n* = 23), and old (32 months old, *n* = 26), after receiving an 8-week period of either progressive resistance protrusive tongue exercise, or sham exercise conditions. Following exercise, HG and SG tongue muscle contractile properties were assessed *in vivo*. HG and SG muscles were then isolated and assayed to determine myosin heavy chain isoform (MyHC) composition.

**Results:** Both retrusive tongue muscle contractile properties and MyHC profiles of the HG and SG muscles were significantly impacted by age, but were not significantly impacted by tongue exercise. Old rats had significantly longer retrusive tongue contraction times and longer decay times than young rats. Additionally, HG and SG muscles showed significant MyHC profile changes with age, in that old groups had slower MyHC profiles as compared to young groups. However, the exercise condition did not induce significant effects in any of the biological outcome measures.

**Conclusion:** In a rat model of protrusive tongue exercise, aging induced significant changes in retrusive tongue muscles, and these age-induced changes were unaffected by the tongue exercise regimen. Collectively, results are compatible with the interpretation that protrusive tongue exercise does not induce changes to retrusive tongue muscle function.

## Introduction

Normal swallowing function requires a series of precisely timed pressure changes within the upper aerodigestive tract that are accomplished primarily by tongue muscle contractile activity, which contributes to bolus containment and propulsion through the upper esophageal segment (Logemann, 1998; Lind, 2003). However, each year, 1 in 25 adults in the United States experience swallowing disorders (dysphagia) (Bhattacharyya, 2014). Prevalence increases with aging and age-related diseases with 50–75% of nursing home residents estimated to have dysphagia (Steele et al., 1997; O’Loughlin and Shanley, 1998), more than 80% of patients with Parkinson disease (Kalf et al., 2012; Suttrup and Warnecke, 2016), and 46–60% of patients with head and neck cancer (Shune et al., 2012). Swallowing impairment is common in elderly individuals (Eisenstadt, 2010), can significantly impact quality of life (Altman et al., 2010; Gassert and Pearson, 2016), and may lead to malnutrition (Altman et al., 2010), aspiration pneumonia (Martino et al., 2005) and dehydration (Smithard et al., 1996; Altman et al., 2010). Age-related changes in muscle composition have been shown to occur in tongue muscles (Rosenberg, 1997; Marzetti and Leeuwenburgh, 2006), with contractile and fiber properties shifting toward a slower contracting fiber profile with increasing age (Connor et al., 2008; Schaser et al., 2011). These changes may contribute to reduced tongue pressures and increased times to reach peak swallowing pressures in elderly persons, thereby increasing risks for dysphagia (Robbins et al., 1992, 1995; Nicosia et al., 2000). Exercise-based treatments to improve swallow function have been developed to target musculature involved in swallowing and deglutition. Since muscles of the tongue are highly active through bolus formation (Kayalioglu et al., 2007), bolus transport and propulsion (Van Daele et al., 2005; Palmer et al., 2008), tongue exercise has frequently been used clinically as a treatment for dysphagia (Robbins et al., 2005; Lazarus, 2006; Park et al., 2015; Rogus-Pulia et al., 2016; McKenna et al., 2017). This exercise involves having patients protrude and press their tongues against a fixed external structure, such as a tongue depressor or in more sophisticated approaches, against a pressure probe as is used in the Iowa Oral Performance Instrument (IOPI)^®^ device (Robbins et al., 2005; Adams et al., 2013).

Tongue exercise paired with a water swallow has been modeled in the rat by our laboratory to study the effects of this intervention on tongue musculature (Connor et al., 2009; Kletzien et al., 2013; Krekeler and Connor, 2016; Schaser et al., 2016), with a degree of experimental precision that is not feasible in human subjects. Because of the predominant role of tongue protrusion in this exercise regimen, initial studies have focused on the genioglossus (GG) muscle, which is responsible for protrusion of the tongue (Bailey et al., 2007; Palmer et al., 2008). The hyoglossus (HG) and styloglossus (SG) are retrusive extrinsic tongue muscles. They receive their innervation from the lateral branch of the hypoglossal nerve (McClung and Goldberg, 2000), and contribute to lingual retraction (Car and Amri, 1987; Baer et al., 1988; Amri et al., 1989; Gilliam and Goldberg, 1995; Thexton et al., 1998; Napadow et al., 1999; Bailey and Fregosi, 2004). These muscles are active during a multitude of physiological functions such as contributions to airway patency, oral transport, swallowing, respiration, and speech (Hiiemae and Palmer, 2003; Kayalioglu et al., 2007; Liu et al., 2007). The relative contributions of retrusive and protrusive extrinsic musculature during protrusive lingual exercise have not been determined (Bailey et al., 2007; Palmer et al., 2008), but there is evidence that both protrusive (GG) and retrusive (SG, HG) tongue muscles are active during all phases of the oropharyngeal swallow (Amri et al., 1989; Napadow et al., 1999). Given that our laboratory’s experimental protrusive exercise paradigm is paired with a water swallow, all extrinsic tongue muscles are likely activated during the course of the regimen to execute lingual press and swallow actions. In the rat model, a progressive resistance tongue exercise regimen has been shown to result in biological changes to the GG, including increased tongue muscle force generative capacity, trends in increasing muscle fiber size (Connor et al., 2009) and changes in myosin heavy chain composition (MyHC) (Kletzien et al., 2013). However, it is not known whether these measures of muscle biology are manifested in retrusive muscles of the tongue following protrusive tongue exercise.

Myosin heavy chain proteins (MyHC) are integral to the molecular contractile apparatus of muscle. There are a variety of myosin heavy chain protein isoforms, each with different functional properties linked to myofiber contractile velocity, contractile force, and ATPase activity. The contractile velocities and ATPase activities of skeletal muscle fibers correlate with the specific MyHC isoforms that they express, such that MyHC 2b is associated with the fastest contraction velocities and greatest ATPase activity, followed by MyHC 2x, MyHC 2a, and finally, MyHC I, which is associated with comparatively slower contraction (Talmadge et al., 1993; Resnicow et al., 2010). Therefore, characteristics of skeletal muscle contraction speeds and fatigue depend in part upon MyHC isoform profiles which can be unique to each muscle type. MyHC isoform profiles may change in response to age and functional demands (Agbulut et al., 2003; Maejima et al., 2005; Yoshii et al., 2008), and therefore can provide a sensitive molecular indicator of how skeletal muscle properties change over time. While aging is generally associated with shifts in MyHC profiles, the nature of these shifts can vary with muscle type, such that some muscles reportedly adopt a faster MyHC profile with aging, while other muscles adopt a slower MyHC profile (Monemi et al., 1999). In the context of tongue muscles specifically, while aging results in slower MyHC profiles of the intrinsic tongue (Cullins and Connor, 2017), exercise may lead to shifts to faster MyHC profiles in some tongue regions (Cullins et al., 2018). Neuromuscular electrical stimulation (NMES) of the hypoglossal nerve has been found to alter HG and SG muscles, resulting in shifts toward slower-contracting MyHC isoform profiles, as well as longer contraction times and reduced muscle fatigue (Kletzien et al., 2018). However, NMES consists of highly controlled increases in tongue muscle activity, and therefore these prior findings may have limited applicability to the question of whether increases of tongue muscle activity through other means, such as voluntary tongue exercise, could similarly impact HG and SG muscles.

Thus, the purpose of this study was to examine the impacts of protrusive tongue exercise on the biology of the extrinsic, retrusive tongue musculature, specifically in the HG and SG muscles. We hypothesized that age would alter HG and SG muscles to slower muscle contraction times, with MyHC isoform profiles of more slowly contracting, fatigue-resistant forms, and that tongue exercise would mitigate these aging effects.

## Materials and Methods

### Protrusive Tongue Exercise

This study was performed in accordance with the NIH Guide for Care and Use of Laboratory Animals (The National Academies, 2011), and the University of Wisconsin School of Medicine and Public Health Animal Care and Use Committee in Madison, Wisconsin (IACUC) approved the animal care and use protocol. In this study, 79 male Fischer 344 Brown Norway rats of 3 different ages [young adult (9 month), middle age (24 month) and old (32 month)] were divided into two groups: exercise and exercise sham (control). Exercise training was conducted on a reverse light cycle, to which rats were acclimated prior to training. Following this, rats were gradually acclimated over the course of a second week to conditions of water regulation, such that free access to water was provided for 3 h each day. Thereafter, rats were trained to use their tongue to press a disk in order to obtain a water reward. The baseline Estimated Maximum Press (EMP, average of the highest voluntary protrusive lingual pressing forces) of each rat was determined and was used to set individualized tongue press force thresholds for subsequent exercise. Rats were trained to elicit the water reward at progressively increased forces, as previously described (Kletzien et al., 2013; Krekeler and Connor, 2016). That is, rats were trained to press at 50% of EMP for two weeks, followed by 60% of EMP for the next two weeks. Midway through the second week of exercise at 60% EMP, revised EMP values were determined for each rat based on midpoint EMP testing. This was followed by two weeks each of exercise at 70% of EMP and 80% of EMP, for a cumulative total of eight weeks of progressive tongue force exercise. During the eight weeks, rats were trained five days per week. For each exercise session, rats provided a minimum of 20 tongue presses per session at or above their respective force threshold during a time period of 10 min. Following this, final EMP testing was completed over a three-day period to determine the final EMP force of each rat. Control rats received experimental conditions and handling that were similar to experimental rats, with the exception that during exercise sessions they were placed in the apparatus and allowed to lick or press briefly with trivial force in order to retain the behavioral task, then were promptly removed from the apparatus. That is, control rats were in the operandum until they did the task each session, but only to the minimum degree needed to keep them acclimated behaviorally to the task. However, control rats and experimental rats received the same procedures for EMP testing.

### Muscle Contractile Properties

Retrusive tongue muscle contractile properties were analyzed as previously described (Becker et al., 2015; Kletzien et al., 2018). Briefly, rats were weighed, then deeply anesthetized with pentobarbitol administered intraperitoneally. Nerve cuff electrodes were placed bilaterally around the hypoglossal nerves. A silk suture was placed in the tip of the tongue, which was manually extended and connected to a force transducer. Optimal line tension on the suture was verified for each rat by adjusting the line tension to elicit the maximal twitch contraction. Hypoglossal nerves were stimulated bilaterally to generate retrusive tongue movements. Stimuli were delivered at 1 Hz to evaluate muscle twitch. Stimulation waveforms were.1-ms rectangular pulses at a supramaximally applied current, defined as 1.5 times the maximum current level required to obtain maximal twitch force (A-M Systems Differential AC amplifier, model 177; A-MSystems Isolated Pulse Stimulator, model 2100; A-M Systems Analog Stimulus Amplification Unit, model 2200, Carlsborg, WA). Data were electronically acquired directly into a dedicated computer using software developed for this purpose (Acquire 1.5; Madison, WI). Maximal twitch contraction time (the duration between the stimulus onset and 50% peak twitch tension), twitch contraction half-decay time (the duration between the stimulus onset and 50% decay from peak twitch tension), and maximal twitch force (the peak tension elicited by a single stimulus) were analyzed. Tetanic forces were elicited through 200 ms trains with stimulus rates of 60, 80, and 100 Hz, and maximal tetanic force (the maximal force of each stimulated fused wave) was recorded. After data collection, rats were euthanized and tissue was isolated for further analysis. Muscle samples were assigned alphanumeric codes to manage risks for unconscious experimental bias in subsequent molecular analyses.

### Sodium Dodecyl Sulfate-Polyacrylamide Gel Electrophoresis

Muscle samples were stored in an −80°C freezer until analysis. As previously described, muscle samples were homogenized and protein concentration was determined using a Bradford Protein Assay. 400 ng protein was analyzed per sample through sodium dodecyl sulfate polyacrylamide gel electrophoresis (SDS-PAGE) comprised of 0.75-mm-thick 6% acrylamide/30% glycerol separating gel (18 cm × 16 cm) and a 4% acrylamide/30% glycerol stacking gel, using the Hoefer SE 600 system (Amersham Biosciences, Piscataway, NJ). The running conditions were set at 275 V, 40 mA, and 15 W for 24 h at 8°C. The gels were stained and developed using Silver Quest Silver Staining Kit (Invitrogen, Carlsbad, CA) to visualize protein bands. As previously described, each MyHC isoform was identified through relative electrophoretic mobility (2a < 2x < 2b < I) (Kletzien et al., 2013). As a control for type I MyHC isoform, we used samples of soleus muscle, a predominately slow twitch muscle in the rat, and for type MyHC2a, MyHC2x, MyHC2b we used samples of extensor digitorum longus (EDL) muscle, a predominately fast twitch muscle in rat.

### Statistical Analysis

One-way Analysis of Variance (ANOVA) was performed to evaluate the impact of age on baseline EMP values at initial tongue press testing. Two-way ANOVAs were performed to determine the impact of independent variables (exercise and age) on the dependent biological measures of estimated maximum press force, myosin heavy chain isoform profiles, decay time, and twitch time. ANOVAs were performed using Graphpad Prism v9 (La Jolla, CA). The measures of maximum tetanic force and maximum twitch force were analyzed using IBM SPSS Statistics v.25, through ANCOVA with body weight as a covariate, in order to accommodate the possibility that body weight influences muscle contraction force measurements (Hurd et al., 2011). The mean body weights (SD) of groups on the day of euthanasia were: Young Adult Control: 406.1 (16.9) g, Young Adult Exercise: 400.5 (20.11) g, Middle Control: 500.3 (43.2) g, Middle Exercise: 498.8 (30.2) g, Old Control: 450.5 (37.49) g, Old Exercise: 448.1 (53.73) g. No data points were removed from analysis, although incidental loss due to technical artifact, health-related attrition or sample loss resulted in unequal group sizes in some experiments. Therefore, of the 79 rats originally assigned to this study, 73 rats ultimately contributed data for this study. Significance was defined at α = 0.05. Statistical tests are summarized in Supplementary Table 1.

## Results

### Protrusive Tongue Exercise

**FIGURE 1:**
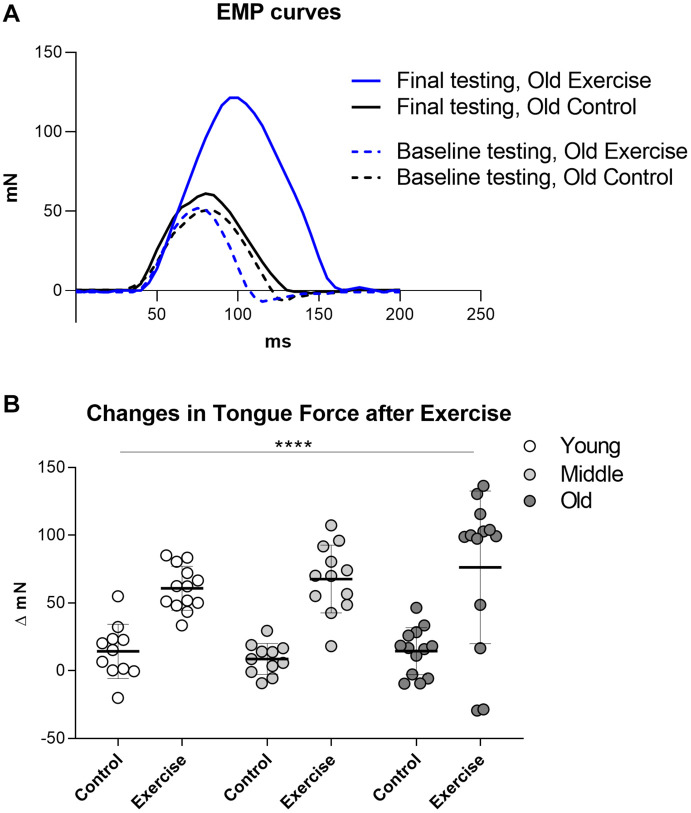
Change in EMP (Estimated Maximum Press) before and after exercise regimen. **(A)** Examples of tongue press force curves before and after tongue exercise in one old control rat and one old exercise rat. **(B)** Quantification of changes in Estimated Maximum Press (EMP) forces between initial and final tongue press testing. Results were significant for exercise condition, but not for age. Each symbol indicates data for one rat. Plots indicate group means and SD. Group sizes: Young Adult Control = 11, Young Adult Exercise = 13, Middle Aged Control = 11, Middle Exercise = 12, Old Aged Control = 13, Old Aged Exercise = 13. **** = *P* < 0.0001.

At baseline, EMP were not significantly different between the age groups of old (Mean = 50.35 mN, SD 15.81), middle (Mean = 56.79 mN SD 19.16) and young (Mean = 53.86 mN SD 14.59). Final EMP forces at the completion of the protrusive tongue exercise regimen were significantly greater in the exercise condition (Mean of all ages = 124.8 mN, SD 36.4) than the control condition (Mean of all ages = 62.88 mN, SD 16.35). Following the protrusive tongue exercise regimen, mean tongue press force increases (delta EMP) of exercise groups were collectively 5.8-fold greater than mean tongue press force increases of control groups which received sham exercise. Separated by age, final EMP forces were 60.78 mN greater than baseline in the young adult exercise group, 67.52 mN greater than baseline in middle aged adult exercise group, and 76.26 mN greater than baseline in the old exercise group. Analyses of changes in tongue force (delta EMP) between the beginning and the end of the 8-week exercise regimen confirmed a highly significant exercise effect (*p* < 0.0001), in the absence of a significant age effect, and in the absence of significant interactions between age and exercise (Figures 1A,B).

### Retrusive Muscle Contractile Properties

**FIGURE 2:**
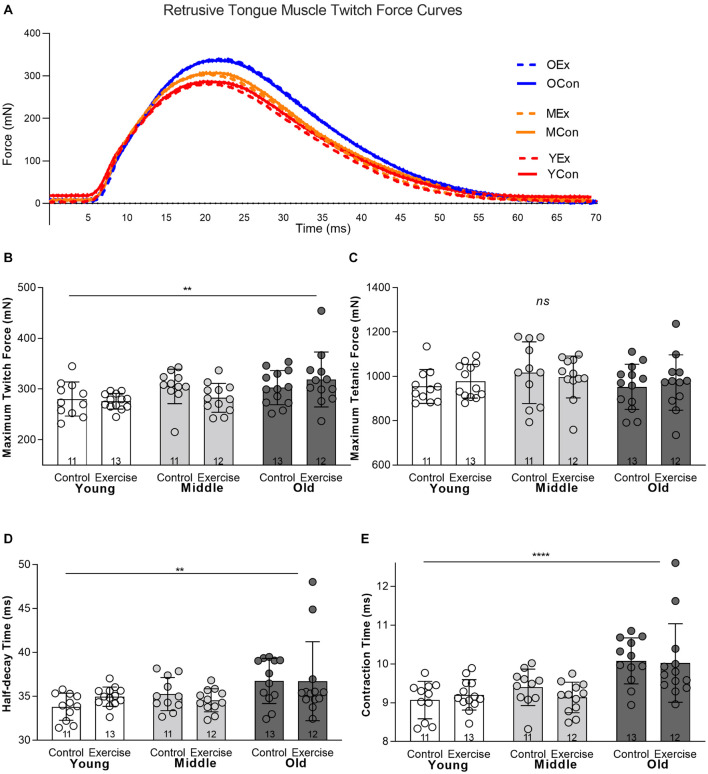
*In vivo* retrusive tongue muscle contractile properties showed significant differences for age, but were not significantly impacted by exercise. **(A)** Example twitch force curves are shown for one individual rat from each of the six experimental groups. YEx = Young adult, Exercise, YCon = Young adult, Control, MEx = Middle aged, Exercise, MCon = Middle aged, Control, OEx = Old age, Exercise, OCon = Old age, Control. **(B)** Maximum Twitch Force **(C)** Maximum Tetanic Force, **(D)** Half-decay Time, **(E)** Contraction Time. Bars show mean and SD, with specific group sample sizes indicated. Each symbol indicates data for one rat. **** = *P* < 0.0001, ** = *P* < 0.01, ns = no significant differences.

There was a significant main effect for age on some measures of retrusive muscle contractile properties. There was not a significant interaction effect between age and exercise, and the exercise condition did not have a significant effect on retrusive muscle contractile properties. Irrespective of the exercise condition, maximum twitch force was significantly greater in the old (Mean = 310.4 mN, SD 44.41) than middle aged (Mean = 293.5 mN, SD 32.55) or young adult groups (Mean = 277.9 mN, SD 24.94) (*p* = 0.01) (Figures 2A,B). However, age had no significant effect on the Maximum Tetanic Force (*p* = 0.116) (Figure 2C). There was a significant main effect for age on half-decay time, such that the old group had longer mean half-decay times (Mean = 36.73 ms, SD 3.62) than middle aged (Mean = 34.89 ms, SD 1.61) or young groups (Mean = 34.44 ms, SD 1.413) (*p* = 0.004) (Figure 2D). There was also a significant main effect for age on muscle contraction time, with the old group having longer mean contraction times (Mean = 10.05 ms, SD .82) than middle aged (Mean = 9.25 ms, SD .44) or young adult groups (Mean = 9.14 ms, SD .43), (*p* < 0.0001) (Figure 2E).

### Myosin Heavy Chain Composition Profiles

**FIGURE 3:**
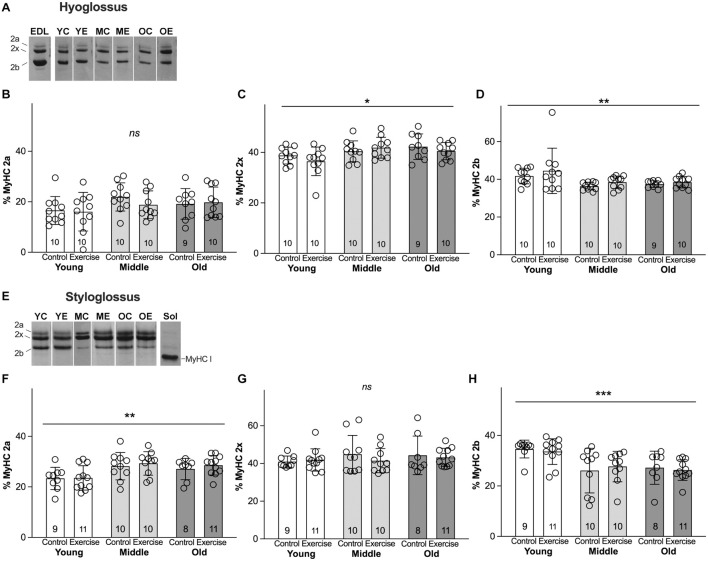
Myosin heavy chain (MyHC) isoform profiles of retrusive tongue muscles change with age, but not with exercise. **(A)** Representative silver-stained hyoglossus (HG) MyHC gel samples. **(B)** HG MyHC 2a. **(C)** HG MyHC 2x. **(D)** HG MyHC 2b. **(E)** Representative silver-stained styloglossus (SG) MyHC gel samples. **(F)** SG MyHC 2a. **(G)** SG MyHC 2x. **(H)** SG MyHC 2b. Sol = Soleus muscle control sample, demonstrating relative mobility of the MyHC I isoform. EDL = Extensor digitorum longus muscle control sample, demonstrating relative mobility of the MyHC isoforms. YC = young control, YE = young exercise, MC = middle control, ME = middle exercise, OC = old control, OE = old exercise. Bars indicate mean and SD. Each symbol indicates data for one rat. Group sizes are indicated in bar graphs. *** = *P* < 0.001, ** = *P* < 0.01, * = *P* < 0.05, ns = no significant differences.

Analysis of MyHC profiles for HG and SG muscles revealed significant main effects for age, and no significant effects for exercise, in the absence of interactions between age and exercise. In the HG muscle, MyHC isoforms showing significant differences across age were MyHC 2x (*p* = 0.013), and MyHC 2b (*p* = 0.004) (Figures 3A–D). The young adult group showed proportionally greater amounts of MyHC 2b (Mean = 43.13%, SD 8.85) than middle aged (Mean = 37.42%, SD 2.911) or old groups (Mean = 38.01%, SD 2.50). In the HG, the young adult group also showed less MyHC 2x (Mean = 37.61%, SD 4.65) than middle aged (Mean = 41.16%, SD 4.09) or old groups (Mean = 41.34%, SD 8.85). In the SG muscle, there were significant increases in the relative proportion of MyHC 2a with age (*p* = 0.001). The SG in the young adult group showed proportionately lower MyHC 2a content (Mean = 23.49%, SD 4.52) than middle aged (Mean = 28.75%, SD 4.95) or old groups (Mean = 27.95%, SD 3.96). There were also significant reductions in relative proportions of MyHC 2b with age, such that the mean MyHC 2b content of SG muscles was greater in the young adult group (Mean = 34.05%, SD 4.37) than middle aged (Mean = 26.87%, SD 7.39) or old adult groups (Mean = 26.65%, SD 5.12) (*p* = 0.0002) (Figures 3E–H). While small levels of MyHC I were present in SG and HG muscles of some rats, relative levels of this isoform were generally scant or undetectable (Figures 3A,E), and were not significantly different between groups.

## Discussion

This work tested the hypothesis that aging would impact biological properties of retrusive tongue muscles in a rat model, and that a protrusive tongue exercise regimen would ameliorate these aging effects. Rats of three different age groups underwent eight weeks of a progressive tongue exercise regimen, after which behavioral tongue forces significantly increased. At the completion of this exercise regimen, retrusive tongue muscle contractile properties were evaluated *in vivo* through whole hypoglossal nerve stimulation, after which the HG and SG muscles were isolated and evaluated for MyHC protein profiles. It was found that although age significantly impacted retrusive tongue contractile properties and MyHC to more slowly contracting profiles, protrusive progressive resistance tongue exercise did not significantly impact either retrusive tongue contractile properties nor retrusive muscle MyHC profiles.

The finding of significant effects of age on retrusive tongue muscle contractile properties and MyHC profiles in the present study is not unexpected in light of several prior reports describing age-related changes in tongue muscles (Kletzien et al., 2013; Cullins and Connor, 2017). As in the present study, a prior study identified significantly longer retrusive contraction times upon hypoglossal nerve stimulation in old rats than for young adult rats (Becker et al., 2015). These age-related differences in muscle contractile function are broadly mirrored by age-related differences in muscle MyHC profiles. Prior studies have reported that with age, the GG muscle shows MyHC shifts indicative of more slowly contracting, fatigue-resistant fiber types, such that the GG of old rats may have relatively greater proportions of MyHC I, and relatively lower proportions of MyHC 2b than young rats (Connor et al., 2013). Similarly, both the HG and SG muscles in the present study demonstrated shifts toward more slowly contracting, fatigue-resistant fiber types with age, such that the retrusive tongue muscles contained lower relative proportions of MyHC 2b in old rats as compared to young rats. These findings are broadly corroborated by earlier studies which also reported significant MyHC changes in the retrusive extrinsic tongue muscles with age (Connor et al., 2013; Kletzien et al., 2018).

The absence of a detectible effect of tongue exercise on retrusive tongue muscle measures is compatible with the interpretation that the progressive resistance tongue exercise regimen may have a high degree of specificity in direct muscular impact. Tongue protrusion is largely accomplished through contraction of the genioglossus muscle, while synergistic contraction of the transverse and verticalis muscles of the intrinsic tongue are believed to result in tongue elongation (McClung and Goldberg, 2000). Tongue retrusion is believed to be accomplished through contraction of the hyoglossus, styloglossus, and the superior and inferior longitudinal intrinsic tongue muscles (McClung and Goldberg, 2000). However, it is also known that co-activation of both muscles of tongue protrusion and muscles of tongue retrusion may be important for a variety of movements and functions (Fregosi and Fuller, 1997; Fuller et al., 1999). While most muscles of the body have bony or cartilaginous origins and insertions, the tongue muscle system is unique in that it is generally understood to function as a muscular hydrostat, such that it likely depends on co-activation of muscles for support and stability in movement to a degree that is not seen in limb muscles (Gilbert et al., 2007). Both intrinsic and extrinsic muscles of the tongue interdigitate and work synergistically to accomplish tongue protrusion, tongue retrusion, and stabilization (Fuller et al., 1999; McClung and Goldberg, 2000; Gilbert et al., 2007), and therefore it is conceivable that some tongue movements may involve concurrent activation of both protrusive and retrusive tongue muscles (Gilbert et al., 2007). Analysis of exercise-induced MyHC changes in discrete tongue muscles is one way through which evidence may be obtained corroborating either the involvement or exclusion of different tongue muscles in specific tongue movements (Cullins et al., 2018). While investigations of impact of lingual exercise on protrusive tongue musculature (genioglossus) have demonstrated mixed findings (Kletzien et al., 2013; Cullins et al., 2018; Krekeler et al., 2020), current findings of unaltered biological measures of retrusive tongue muscles following tongue exercise suggest that progressive resistance protrusive tongue exercise does not significantly impact the extrinsic muscles of tongue retrusion. These findings suggest that inducing biological change *via* oromotor exercise may require highly specific movements, or practice geared toward activating muscular groups required for the desired action.

There are limitations to this study. While rat models of oromotor functions are invaluable for translational research (German et al., 2017), it should be noted that some intrinsic differences in tongue function between rats and humans may impose certain limitations on the translational applicability of this study. Depending on the species and the context, the ways mammals use the tongue on a daily basis can vary in the magnitude of protrusive tongue movements and retrusive tongue movements (Olson et al., 2021). Notably, while humans typically drink with their tongue positioned intraorally (Matsuo and Palmer, 2008), rats typically drink by protruding the tongue from the oral cavity while licking or lapping (Weijnen, 1998). This constitutes a difference in the utility and magnitude of tongue protrusion and retrusion in rats as compared to humans. Further, while the tongue exercise paradigm studied here involves protrusion of the tongue from the oral cavity to exert progressive force on an external object, tongue exercise regimen in humans are often likely to involve exercises with intraoral tongue movements (Yoshikawa et al., 2021). Therefore, it is possible that rats and humans may be anticipated to differ somewhat in the degree to which retrusive tongue muscles are impacted by a given tongue exercise regimen. These limitations notwithstanding, the biological investigations of discrete tongue muscles that are possible in rat models can provide anatomically precise information that cannot be obtained from humans.

This current study’s findings that protrusive tongue exercise does not impact aging effects in retrusive tongue muscles suggests two implications. First, there is interest in the possibility that tongue exercise regimen can impact or improve swallow function (Kim et al., 2017; Krekeler et al., 2020), although mechanisms through which that may occur remain somewhat unclear. It is known that protrusive tongue muscles play important roles in the oral stage of swallowing, and therefore increased strength or altered function of protrusive muscles may be a means through which tongue exercise could produce improved outcomes (Kim et al., 2017), conceivably in the absence of increased strength or altered function of retrusive tongue muscles. Secondly, these results also inspire the question of whether future experimental work in therapeutic tongue exercise regimen could be modified to target retrusive muscles. It is known that retrusive tongue muscles are integral to retrograde bolus propulsion during swallowing (Gilbert et al., 2007). Although the protrusive tongue exercise task in the current study coincided with a water swallow, modulation of the retrusive movements involved in swallowing was not a direct focus of the regimen. Future investigations using rat paradigms should examine requirements for inducing biological change in targeted muscular groups that will contribute to optimize functional improvements in deglutition and swallowing, including mechanisms of adaptive responses to exercise dose, and fatigue prevention.

## Data Availability Statement

The original contributions presented in the study are included in the article/Supplementary Material, further inquiries can be directed to the corresponding author.

## Ethics Statement

The animal study was reviewed and approved by University of Wisconsin School of Medicine and Public Health Animal Care and Use Committee in Madison, Wisconsin (IACUC).

## Author Contributions

NC conceived of the study and provided the requisite resources. JF, TG, and JR performed experiments in the study. TG, JF, and JR compiled and analyzed the data. JF, TG, JR, BK, and NC wrote and edited the manuscript. All authors contributed to the article and approved the submitted version.

## Conflict of Interest

The authors declare that the research was conducted in the absence of any commercial or financial relationships that could be construed as a potential conflict of interest.

## Publisher’s Note

All claims expressed in this article are solely those of the authors and do not necessarily represent those of their affiliated organizations, or those of the publisher, the editors and the reviewers. Any product that may be evaluated in this article, or claim that may be made by its manufacturer, is not guaranteed or endorsed by the publisher.
