# Supplementary material for: Progressive Protrusive Tongue Exercise Does Not Alter Aging Effects in Retrusive Tongue Muscles
Source: Front Physiol. 2021 Oct 21;12:740876. doi: 10.3389/fphys.2021.740876 (PMC8567011; doi:10.3389/fphys.2021.740876)
Supplement: Supplementary file 1 [file Table_1.DOCX]

**Table S1.** Dependent variables, main effects, interaction effects, f & p-values. Abbreviations: EMP= Estimated Maximum Press, MyHC= Myosin heavy chain isoform, mN= millinewtons, ms= milliseconds, %= percentage of the isoform that comprises total MyHC.

| **Variable** | **Unit** | **Age** | **Exercise** | **Interaction**  **(Age x Exercise)** |
| --- | --- | --- | --- | --- |
| Estimated Maximum Press (EMP) at baseline | mN | F (2, 70) = 0.893  P=0.414 | N/A | N/A |
| Delta EMP | mN | F (2, 67) = 0.562  P=0.573 | F (1, 67) = 66.30  P<0.0001 | F (2, 67) = 0.470  P=0.627 |
| Tongue muscle retrusive contraction time | ms | F (2, 66) = 16.48  P<0.0001 | F (1, 66) = 0.0003  P=0.986 | F (2, 66) = 0.799  P=0.454 |
| Tongue muscle retrusive half-decay time | ms | F (2, 66) = 6.138  P=0.004 | F (1, 66) = 0.398  P=0.530 | F (2, 66) = 0.874  P=0.422 |
| Tongue muscle retrusive maximum tetanic force | mN | F (2,65) = 2.230  P=.116 | F (1,65) = .094  P=.760 | F ( 2, 65) = .375  P=.688 |
| Tongue muscle retrusive maximum twitch force | mN | F (2,65) = 4.901  P=.010 | F (1,65) = 0.223  P=.639 | F (2,65) = 1.656  P=.199 |
| Hyoglossus MyHC 2a | % | F (2, 53) = 2.436  P=0.097 | F (1, 53) = 0.392  P=0.534 | F (2, 53) = 0.480  P=0.621 |
| Hyoglossus MyHC 2x | % | F (2, 53) = 4.702  P=0.0132 | F (1, 53) = 0.570  P=0.454 | F (2, 53) = 1.043  P=0.360 |
| Hyoglossus MyHC 2b | % | F (2, 53) = 6.151  P=0.004 | F (1, 53) = 1.722  P=0.195 | F (2, 53) = 0.122  P=0.886 |
| Hyoglossus  MyHC I | % | F (2, 53) = 3.078  P=0.054 | F (1, 53) = 0.014  P=0.9065 | F (2, 53) = 0.048  P=0.954 |
| Styloglossus MyHC 2a | % | F (2, 53) = 7.438  P=0.001 | F (1, 53) = 0.614  P=0.437 | F (2, 53) = 0.095  P=0.910 |
| Styloglossus MyHC 2x | % | F (2, 53) = 0.569  P=0.570 | F (1, 53) = 0.413  P=0.523 | F (2, 53) = 0.454  P=0.638 |
| Styloglossus MyHC 2b | % | F (2, 53) = 9.961  P=0.0002 | F (1, 53) = 0.047  P=0.946 | F (2, 53) = 0.314  P=0.732 |
| Styloglossus MyHC I | % | F (2, 53) = 2.522  P=0.090 | F (1, 53) = 2.064  P=0.157 | F (2, 53) = 0.953  P=0.392 |
